# Mechanisms of mGluR‐dependent plasticity in hippocampal area CA2

**DOI:** 10.1002/hipo.23529

**Published:** 2023-03-27

**Authors:** Mahsa Samadi, Claire A. Hales, Daniel J. Lustberg, Shannon Farris, Madeleine R. Ross, Meilan Zhao, John R. Hepler, Nicholas H. Harbin, Emma S. J. Robinson, Paul J. Banks, Zafar I. Bashir, Serena M. Dudek

**Affiliations:** ^1^ School of Physiology, Pharmacology and Neuroscience, Biomedical Sciences Building University Walk, University of Bristol Bristol UK BS8 1TD; ^2^ Neurobiology Laboratory, National Institute of Environmental Health Sciences (NIH) 111 T.W. Alexander Drive, Research Triangle Park Durham North Carolina 27709 USA; ^3^ Department of Pharmacology and Chemical Biology Emory University School of Medicine 100 Woodruff Circle Atlanta Georgia 30322 USA; ^4^ Present address: Faculty Education Office, Faculty of Medicine, Imperial College London, Hammersmith Campus, Wolfson Education Centre London UK W12 0NN; ^5^ Present address: Department of Psychology, Djavad Mowafaghian Centre for Brain Health University of British Columbia 2215, Wesbrook Mall Vancouver British Columbia V6T 1Z3 Canada; ^6^ Present address: Mouse Pharmacology Group Psychogenics Inc 215 College Road Paramus New Jersey 07652 USA; ^7^ Present address: Fralin Biomedical Research Institute at Virginia Tech Roanoke Virginia 24014 USA

**Keywords:** hippocampus, long‐term depression, RGS14, social recognition memory, synaptic plasticity

## Abstract

Pyramidal cells in hippocampal area CA2 have synaptic properties that are distinct from the other CA subregions. Notably, this includes a lack of typical long‐term potentiation of *stratum radiatum* synapses. CA2 neurons express high levels of several known and potential regulators of metabotropic glutamate receptor (mGluR)‐dependent signaling including Striatal‐Enriched Tyrosine Phosphatase (STEP) and several Regulator of G‐protein Signaling (RGS) proteins, yet the functions of these proteins in regulating mGluR‐dependent synaptic plasticity in CA2 are completely unknown. Thus, the aim of this study was to examine mGluR‐dependent synaptic depression and to determine whether STEP and the RGS proteins RGS4 and RGS14 are involved. Using whole cell voltage‐clamp recordings from mouse pyramidal cells, we found that mGluR agonist‐induced long‐term depression (mGluR‐LTD) is more pronounced in CA2 compared with that observed in CA1. This mGluR‐LTD in CA2 was found to be protein synthesis and STEP dependent, suggesting that CA2 mGluR‐LTD shares mechanistic processes with those seen in CA1, but in addition, RGS14, but not RGS4, was essential for mGluR‐LTD in CA2. In addition, we found that exogenous application of STEP could rescue mGluR‐LTD in RGS14 KO slices. Supporting a role for CA2 synaptic plasticity in social cognition, we found that RGS14 KO mice had impaired social recognition memory as assessed in a social discrimination task. These results highlight possible roles for mGluRs, RGS14, and STEP in CA2‐dependent behaviors, perhaps by biasing the dominant form of synaptic plasticity away from LTP and toward LTD in CA2.

## 1 INTRODUCTION

Hippocampal area CA2 has been shown to have synaptic properties distinguishing it from the other hippocampal subfields (Dudek et al., 2016). Notably, stimulation protocols that typically induce long‐term potentiation (LTP) in CA1 are ineffective in the CA2 *stratum radiatum* (SR), likely representing the Schaffer collateral synapses (Zhao et al., 2007). Induction of long‐term depression (LTD) in the rat CA2 SR, induced by low frequency stimulation, was similarly less than in CA1, but the comparative deficit varied in that some cells in CA2 showed “normal” LTD and others did not (Zhao et al., 2007). However, the capacity for metabotropic glutamate receptor‐dependent LTD (mGluR‐LTD) in CA2 has not yet been investigated. Though mGluRs are expressed throughout hippocampal dendritic trees, they not markedly enriched in area CA2 (Fotuhi et al., 1993; Lein et al., 2007; Romano et al., 1995; Shigemoto et al., 1997). However, mGluR‐related signaling may be different there compared with CA1. Specifically, mGluR‐stimulated [^3^H]cytidine diphosphate diacylglycerol accumulation appears to be localized in and around area CA2 (Hwang et al., 1990). In addition, many proteins linked to both NMDA and Group I mGluR‐dependent plasticity have been shown to be highly expressed in CA2 (Farris et al., 2019; Gerber et al., 2019). For example, the Striatal‐Enriched Tyrosine Phosphatase (STEP; *Ptnp5*) is highly expressed in CA2 (Boulanger et al., 1995) and is crucial for AMPA receptor internalization following application of the Group I mGluR agonist (R)(*S*)‐3,5‐Dihydroxyphenylglycine (DHPG) in CA1 (Zhang et al., 2008). Thus, mGluR‐dependent plasticity in CA2 may be differentially regulated from that in CA1.

Another protein highly expressed in CA2 is Regulator of G‐protein Signaling 14 (RGS14) (Evans et al., 2014; Lee et al., 2010). RGS14 belongs to a diverse family of RGS proteins that generally limit the activity of G‐Protein Coupled Receptors (GPCRs) by serving as GTPase activating proteins (GAPs) to negatively regulate the activity of Gα subunits (Masuho et al., 2020; Stewart & Fisher, 2015; Vellano et al., 2011). RGS14 differs from the other members of the RGS protein family in that it is a multifunctional scaffolding protein that integrates conventional G‐protein signaling with Extracellular Signal‐Regulated Kinase/Mitogen Activated Protein Kinase (ERK/MAPK) pathways though its tandem Ras binding domains (Shu et al., 2010; Vellano et al., 2013). Remarkably, CA2 pyramidal cells from RGS14 knockout (KO) mice can readily express LTP, unlike those from wildtype (WT) mice (Lee et al., 2010). The exact mechanism(s) by which RGS14 modulates the synaptic properties of CA2 are uncertain, but evidence suggests that RGS14 modulates both ERK and calcium signaling in postsynaptic spines (Evans, Parra‐Bueno, et al., 2018; Harbin et al., 2021). Of note, RGS14 may also act in the nucleus apart from any synaptic functions (Branch & Hepler, 2017; Squires et al., 2021). Another RGS protein, RGS4, is also highly enriched in CA2 (Lein et al., 2007) and has been shown to negatively regulate Group I mGluR‐Gq signaling (Saugstad et al., 1998). Thus, either of these RGS proteins could modulate Group I mGluR‐dependent plasticity in CA2 in a way that makes it distinct from synaptic plasticity in CA1.

In this study, we investigated the nature of mGluR‐dependent synaptic plasticity in CA2 and the roles of STEP, RGS4, and RGS14 in regulating its expression. Using whole cell voltage‐clamp recordings from mouse pyramidal cells, we found that mGluR agonist‐induced long‐term depression (mGluR‐LTD) is more pronounced in CA2 compared with that observed in CA1. This mGluR‐LTD in CA2 was found to be protein synthesis and STEP dependent, suggesting that CA2 mGluR‐LTD shares mechanistic processes with those seen in CA1. In addition, we found that RGS14, but not RGS4, was essential for mGluR‐LTD in CA2. We also found that exogenous application of STEP could rescue mGluR‐LTD in RGS14 KO slices. Finally, supporting a role for CA2 plasticity in social behavior and cognition, we found that RGS14 KO mice exhibited impaired social memory as assessed in a social discrimination task. These studies provide further insight into how plasticity and CA2‐dependent behaviors are regulated in area CA2. Disruption of CA2 plasticity may contribute to social memory impairments in RGS14 KO mice, with possible relevance to human developmental disorders characterized by impairments in social cognition.

## 2 METHODS

All animals were housed under a 12:12 light/dark cycle with access to food and water ad libitum. All procedures carried out at the University of Bristol were in accordance with the UK Animals (Scientific Procedures) Act of 1986 and approved by the University of Bristol Animal Welfare and Ethical Review Body. All procedures carried out at NIEHS were approved by the NIEHS Animal Care and Use Committee.

### 2.1 Electrophysiology

Hippocampal slices were prepared from 13 to 21 day old (P13‐21) RGS14 knockout (KO), RGS4 KO, STEP KO, or WT control (C57BL/6J) mice. RGS14 KO mice (B6;129S5‐Rgs14tm1^Lex/Mmnc^) were obtained via Prof. John Hepler (Emory University, Atlanta; Lee et al., 2010) and bred at both NIEHS, USA and University of Bristol, UK. RGS4 KO mice (B6;129P2‐Rgs4^tm1Dgen/J^) were obtained from the Jackson Laboratory (USA). STEP KO mice (B6N.129‐Ptpn5^tm1Pijlo/J^) were originally from Prof Paul Lombroso (University of Yale, New Haven, CT) and bred at the University of Bristol, UK. No more than one cell per condition was recorded per animal (*n* cells = *n* animals). The experimenter was not blinded to genotype.

Mice were deeply anesthetized with isoflurane (Bristol) or pentobarbital (NIEHS) and decapitated. Their brains were then rapidly removed and placed in an ice‐cold sucrose cutting solution: (in mM) 189 sucrose, 10 d‐glucose, 26 NaHCO_3_, 3 KCl, 5 MgSO_4_7H_2_O, 0.1 CaCl_2_, 1.25 NaH_2_PO_4_, and equilibrated with 95% O_2_/5% CO_2_. The brain was hemisected along the midline and the midline surface mounted with cyanoacrylate glue. Parasagittal slices (300 μm) from the lateral side of either hemisphere were prepared using a ceramic or metal blade on a vibrating microtome (7000 smz, Camden instruments, Loughborough (Bristol) or Leica VT1200S (NIEHS), respectively) with an advance speed of 0.1 mm/s. As slices were collected, they were placed into a holding chamber containing artificial cerebral spinal fluid (ACSF) kept in a water bath held at ~35°. After being allowed to rest for 30 min, slices were kept at room temperature for the remainder of the recovery period (minimum 30 min) before being transferred to the recording chamber. ACSF (in mM): 124 NaCl, 2.5 KCl, 2 MgCl_2_, 2 CaCl_2_, 1.25 NaH_2_PO_4_, 26 NaHCO_3_, and 17 d‐glucose, and was equilibrated with 95% O_2_/5% CO_2_.

Slices from the dorsal portion of the hippocampus were submerged in a chamber and continuously perfused with room temperature ACSF at a flow rate of 2 mL/min. Recordings were performed at room temperature because CA2 neurons, relative to those in CA1, are more difficult to hold for the length of time required for these experiments when at higher temperatures. All patch clamp recordings were made in voltage clamp mode held at −70 mV using patch pipettes fabricated from borosilicate glass capillaries pulled using a horizontal puller (P‐97, Sutter Instruments), with a resistance of 2 to 5 MΩ. Pipettes were filled with a cesium (Cs) based internal solution containing the following component (in mM): 115 Cs‐methanesulfonate, 20 CsCl, 5 MgCl_2_, 0.6 EGTA, 10 HEPES, 4 Na_2_‐ATP, 0.4 Na‐GTP, and 10 phosphocreatine disodium salt, with an osmolarity of 280–300 mOsm. CA2 pyramidal cells were identified by visual appearance and anatomical location. Responses were evoked every 30 s using afferent stimulation (0.1 ms pulse duration) applied to the Schaffer Collateral inputs via a 2‐contact cluster electrode (FHC, USA) placed in the *stratum radiatum* (SR). Stimulating electrodes in the SR were always placed away from the pyramidal cell layer to reduce the likelihood of stimulating dentate gyrus mossy fibers. Experiments were focused on the SR because preliminary studies in rats had shown that synaptic responses evoked by stimulation in the *stratum lacunosum moleculare* had a smaller DHPG‐induced depression than that seen in responses evoked in SR (data not shown). Bath application of GABA antagonists increased the incidence of epileptiform activity, and so we did not use them in our LTD experiments. However, in a separate series of recordings, we observed a 22.5% reduction in the amplitude of synaptic responses at −70 mV upon wash in of the GABA antagonists (1 μM SR 95531 hydrobromide and 2 μM CGP 55845 hydrochloride to block GABAa and GABAb receptors, respectively; *n* = 7). Therefore, because no attempt was made to determine how much of the synaptic currents were excitatory in each individual experiment, we designated them as Post‐Synaptic Currents (PSCs) instead of Excitatory PSCs as they represent mixed excitatory and inhibitory synaptic responses. We note though, that DHPG has now been shown to induce LTD in CA2 field potential recordings, even when inhibition is blocked (Loisy et al., 2022).

Data were acquired using an Axopatch 200B amplifier (Molecular Devices) and WinLTP software, filtered at 2 kHz and digitized at a sampling rate of 20 kHz. Using either the M,X series (National Instruments, UK; Bristol) board or the Digidata 1322A (Molecular Devices, NIEHS). Data from experiments with >20% change in series resistance were discarded due to possible effects on response amplitude.

All compounds used for pharmacological experiments were from either Tocris or Abcam and prepared as stock solutions in either distilled water or dimethyl sulfoxide (DMSO), stored at −20°C and then freshly prepared to the desired concentration in ACSF before each experiment. Experiments with ACSF/DMSO (0.1%) as a vehicle control were performed to ensure the maximum concentration used in experiments did not produce any undesired effect. In a subset of experiments using RGS14 KO slices, exogenous active or inactive STEP (GST STEP46 WT, purified and generously provided by the Lombroso lab) was included in the intracellular solution (diluted ~1000 fold for final concentrations of 5 μg/mL STEP and ~50 μM Tris–HCl).

#### 2.1.1 Immunofluorescence

Mice (P21 C57BL/6J; Charles River) were deeply anesthetized with sodium pentobarbital (Fatal‐Plus, 100 mg/kg) before being perfused with chilled 4% paraformaldehyde diluted in ×1 phosphate buffered saline (PBS). Brains were removed and stored overnight at 4°C in 4% paraformaldehyde diluted in ×1 PBS for post‐fixation. Brains were then transferred to ×1 PBS supplemented with 0.01% sodium azide until sectioning. Sections 40‐μm thick were cut on a vibratome (Leica VT1200 S) and stored at 4°C in ×1 PBS with 0.01% sodium azide before immunohistochemistry.

Sections were washed in ×1 PBS (×2 for 15 min) and 0.1% phosphate buffered saline with 0.1% Triton‐X (PBST; ×1 for 15 min). Sections were then blocked with 5% normal goat serum (NGS, Vector Laboratories, S‐1000) diluted in 0.1% PBST either overnight at 4°C—for total STEP and active, non‐phosphorylated STEP stains—or for 1 h at room temperature (RT). Sections were next incubated overnight rocking at 4°C in primary antibody solution prepared with 5% NGS/0.1% PBST. Primary antibodies were: mouse anti‐STEP (1:500, Cell Signaling Technology, 4396), rabbit anti‐non‐phospho‐STEP (1:500, Cell Signaling Technology, 5659), mouse anti‐RGS14 (1:500, NeuroMab, 75‐170), and/or rabbit‐CaMKII alpha (1:500, Abcam, ab131468). Sections were washed in 0.1% PBST (×3 for 10 min) before incubation for 2 h at RT in secondary antibody solution prepared with 5% NGS/0.1% PBST. Secondary antibodies were: AlexaFluor 488 goat anti‐mouse IgG1 (1:500, Invitrogen, A21121) for total STEP, AlexaFluor 488 goat anti‐rabbit IgG (1:500, Invitrogen, A11034) for non‐phospho‐STEP and CaMKII alpha, and AlexaFluor 555 goat anti‐mouse IgG2a (1:500, Invitrogen, A21137) for RGS14. Sections were then washed again in 0.1% PBST (×3 for 10 min) and left rocking overnight at 4°C in ×1 PBS before being mounted on Superfrost Plus slides (Fisher Scientific) using Vectashield HardSet antifade mounting media containing DAPI (Vector Laboratories, H‐1500). Z‐stack maximum intensity projections and single plane micrographs were acquired under standardized exposure parameters at ×20 and ×40 using a Zeiss LSM 880 inverted confocal microscope. Images were adjusted for brightness with ImageJ for presentation purposes (Schneider et al., 2012) and so are not intended for comparisons.

### 2.2 Proximity ligation assay

For measurement of likely protein–protein interactions, we used the Duolink® In Situ starter kit for rabbit/mouse (Sigma‐Aldrich, USA, No. DUO92101) per the manufacturer's instructions, which were based on the technology reported by Fredriksson et al. (2002). Free‐floating coronal sections containing dorsal hippocampus were placed in tubes containing 10 mM sodium citrate buffer and submerged in boiling water for 3 min for antigen retrieval. Sections were then incubated in the blocking solution provided in the kit (donkey serum) for 2 h at room temperature. Primary antibodies were diluted in the kit's blocking serum at the following concentrations: anti‐mouse RGS14 (1:500 Neuromab), anti‐rabbit CaMKII (1:250, Abcam, No. 131468), anti‐mouse Actin (1:1000, Synaptic Systems, Germany, No. 251011), anti‐rabbit Arc (1:1000, Synaptic Systems, No. 156003), and anti‐mouse NeuN (1:500, Millipore, No. ZMS377). Sections were incubated overnight in the following combinations: RGS14 and CaMKII; RGS14 alone; CaMKII alone, Actin and Arc for a positive control, and NeuN and Arc for negative control. Sections were then washed the next day in the kit's wash buffer (3× 5 min). The complementary mouse and rabbit proximity ligation assay (PLA) probes were diluted 1:5 with the buffer provided in the kit and applied to the samples for 60 min at 37°C. Slices were then washed in ×1 wash buffer A provided in the kit (2× 5 min). For ligation, the ligation stock provided was diluted 1:5 in the kit's diluent, which was also used to prepare the ligase at 1:40 in the solution. The amplification reagent was prepared at 1:5 in distilled water. Following completion of the protocol and tissue mounting, images were acquired on Zeiss 710 confocal microscope at ×63 oil objective, using the same 561 laser power and z‐stack acquisition parameters throughout.

### 2.3 Behavior

Mice were housed 4 per cage with ad lib access to food and water. Each cage consisted of 2 WT and 2 RGS14 KO mice so that behavioral tests could be conducted using both genotypes as familiar and novel test subjects. From the age of weaning, mice were kept on a 12 h reversed light–dark cycle (lights off at 8.15 AM). Three days before testing, mice were habituated to the handling and the transport to the behavioral room and apparatus. On day 1, mice were taken to the behavior room in their housed cages for 1 h. On day 2, mice were placed in the behavioral apparatus for 5 min in their housed groups of 4. On day 3, mice were individually placed in the behavioral apparatus for 5 min to complete the habituation process. Experimental testing began on day 4. To blind the experimenter to genotype and to ensure littermates were never used in the same experiment, each litter and cage were given a color and number to identify individual littermates and cagemates. The genotype of each mouse was revealed only after all the analyses were performed. Animals used in this study were between 2 and 3 months of age.

To test the role of RGS14 on social recognition memory, RGS14 WT or RGS14 KO mice were placed for 5 min in a transparent Perspex arena (40 cm length × 20 cm width × 20 cm height) which contained two inverted wire cups (3.8 cm bottom diameter) placed on either side of the arena, with sufficient space for the test mouse to approach from any side of the wire cup. A familiar (cagemate) or novel mouse was placed into each wire cup ahead of the test session and the test mouse was then placed in the middle of the arena and was left to explore for 5 min. The mesh of the wire cup allowed for social interaction using visual, olfactory, acoustic, and tactile signals, but not direct physical contact. A weighted jar was placed on top of each wire cup to prevent the test subject mouse from climbing and to prevent any movement of the wire cup once positioned. The location of the familiar and novel mice was alternated between test sessions to prevent any biasing from external cues and to keep the experimenter blinded to the identity of the mice. After each 5‐min test session, the arena, wire cups, and weighted jars were all thoroughly cleaned with 70% ethanol to remove any olfactory cues before the next test session took place. The social interaction time (defined as active sniffing of, or interacting with, the mouse inside the wire cup) was measured in a 5‐min timeframe and recorded using a Logitech c920 webcam. Scoring was carried out by experimenters blinded to genotypes of both the test mouse and the social cue mouse. The discrimination ratio was then calculated by dividing the difference in interaction time (total novel time – total familiar time) by the total interaction time (total novel time + total familiar time). One mouse was excluded from the analysis due to abnormal home cage and test arena aggression.

### 2.4 Statistics

For slice experiments, data were analyzed using SPSS software. Control data were pooled and tested for normal distribution using the Shapiro–Wilk test. From this, it was assumed that all datasets followed a normal distribution and were therefore analyzed using parametric testing. If main effects were significant, subsequent post hoc paired sample *t*‐tests were carried out with raw values to compare PSC amplitude before and after drug application or stimulation protocol. Normalized values were used for comparisons between CA2 and other hippocampal subfields as well as WT and KO animals. SigmaPlot was used for graphical representation.

For the social recognition memory behavior, discrimination ratio scores and total interaction time were analyzed with independent samples *t*‐tests to compare genotypes. Discrimination ratio scores were also analyzed with one sample *t*‐tests to test whether mice were able to discriminate between novel and familiar conspecifics. Statistics were conducted using SPSS 24.0.0.2 for Windows (IBM SPSS Statistics) with *α* = 0.05. Results are reported with the *T*‐statistic (degrees of freedom) and *p*‐value. Graphs and images were made using Graphpad Prism 7.04 for Windows (Graphpad Software, USA) and Python 3's matplotlib package (Python 3.9.6 for Windows).

## 3 RESULTS

### 3.1 DHPG induces robust LTD in CA2: Roles of RGS14 and RGS4

**FIGURE 1:**
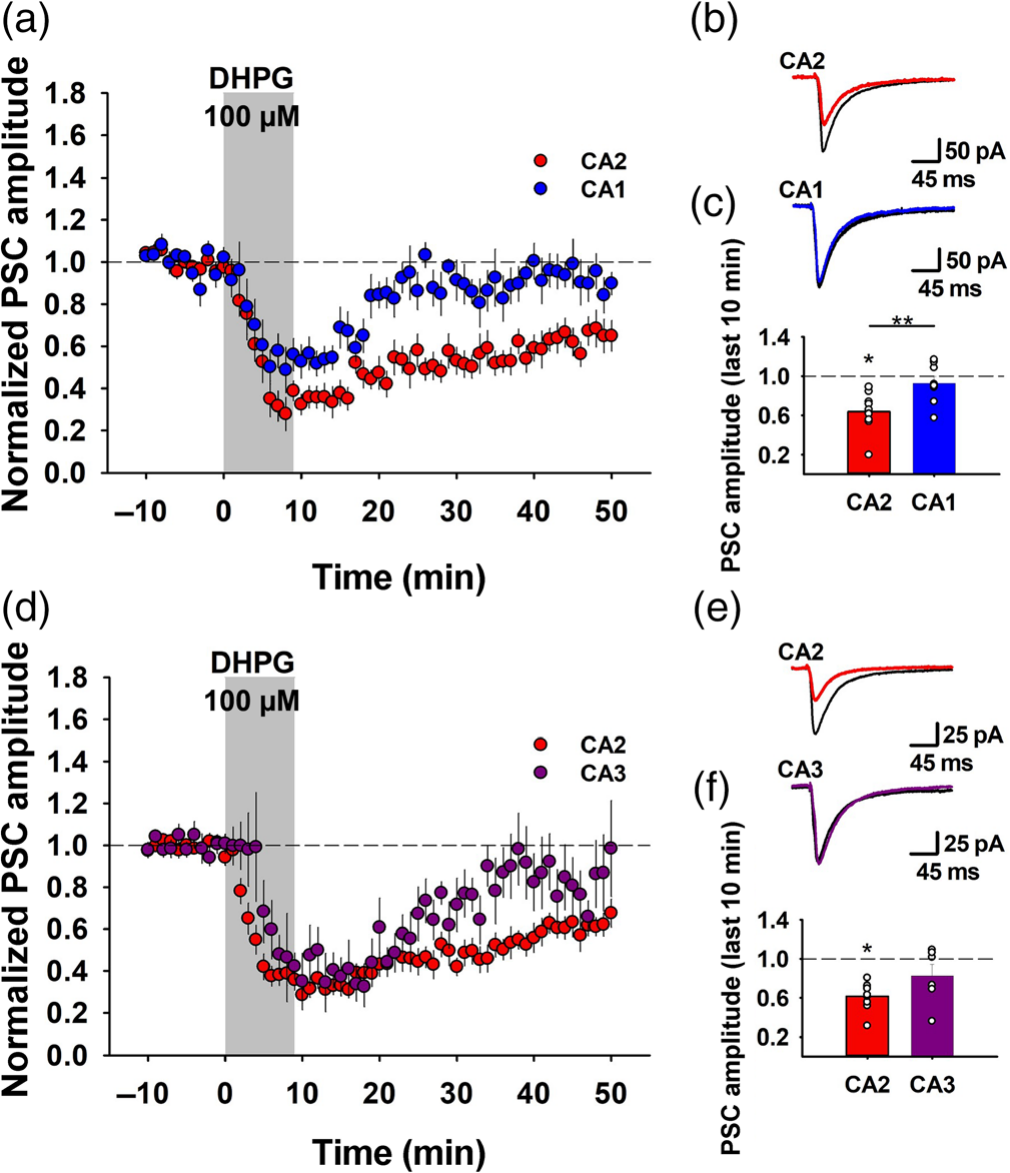
DHPG induces LTD in CA2 in P13‐P21 mice. (a) Normalized PSC amplitudes recorded in CA2 or CA1 following 100 μM DHPG application. Data points represent the mean PSC amplitude. Error bars represent the standard error of the mean. Red circles represent recordings from CA2 pyramidal cells (*n* = 10). Blue circles represent recordings from CA1 (*n* = 8). Gray shaded area indicates duration of DHPG application. (b) Example PSC traces from CA2 and CA1 pyramidal cells before and 40 min after completion of DHPG application. Black traces represent PSC response before DHPG application. Above: red trace indicates PSC response of CA2 after DHPG application. Below: blue trace represents PSC response of CA1 after DHPG application. (c) Normalized PSC amplitude of CA2 (red) and CA1 (blue) cells at 41–50 min during recovery. Asterisk denotes significant difference (*p* ≤ .05) when compared with mean PSC amplitude before DHPG application. Double asterisk denotes significant difference in PSC amplitude when comparing CA2 and CA1 pyramidal cells. (d) Normalized PSC amplitude recorded in CA2 or CA3 following 100 μM DHPG application. Red circles represent recordings from CA2 pyramidal cells (*n* = 9). Magenta circles represent recordings from CA3 (*n* = 6). (e) Example PSC traces from CA2 and CA3 pyramidal cells before and 40 min after completion of DHPG application. Black traces represent PSC response before DHPG application. Above: red trace indicates PSC response of CA2 after DHPG application. Below: Magenta trace represents PSC response of CA3 after DHPG application. (f) Normalized PSC amplitude of CA2 (red) and CA3 (magenta) cells at 41–50 min during recovery.

Previous work has demonstrated that inhibition of Group III mGluRs promotes LTP in CA2 (Dasgupta et al., 2020); however, little is known about how Group I mGluRs might regulate synaptic plasticity in CA2. To determine whether group I mGluR activation causes synaptic depression as reported in CA1 (Huber et al., 2001), we applied the group I mGluR agonist (R)(*S*)‐3,5‐dihydroxyphenylglycine (DHPG) to acutely prepared hippocampal slices while recording synaptic responses from CA2 pyramidal neurons of P13‐P21 mice. We found that a 10‐min application of 100 μM DHPG induced a depression of the synaptic responses lasting at least 50 min (0.63 ± 0.06 PSC amplitude normalized to baseline, *n* = 10; Figure 1a,b). Under our recording conditions (i.e., room temperature); however, DHPG application caused no significant lasting change of PSC amplitude in CA1 pyramidal cells (0.93 ± 0.07, *n* = 8; Figure 1a,b). Paired *t*‐tests of raw PSC amplitude at baseline (10 min before DHPG application) and recovery (40–50 min after DHPG was first applied) revealed a significant change in PSC amplitude of CA2 (*t* = −5.565, *p* ≤ .001) but not CA1 pyramidal cells (*t* = 0.133, *p* = .898). Comparison of normalized PSC amplitude after recovery revealed a significant difference in PSC amplitude between CA2 and CA1 pyramidal cells following DHPG application (*t* = −3.427, *p* = .003; Figure 1c). Similarly, in a limited number of CA3 neurons, DHPG failed to induce a lasting significant change in PSC amplitude (0.81 ± 0.12, *n* = 6; Figure 1d,e), but again produced depression of PSPs in CA2 pyramidal cells in a cohort of animals different from the CA1 experiments (0.63 ± 0.05, *n* = 9; Figure 1f). Paired *t*‐tests comparing raw PSC amplitude at baseline and recovery again revealed a significant difference in PSC amplitude of CA2 pyramidal cells after DHPG application (*t* = −6.453, *p* ≤ .001), but not when comparing PSC amplitude of CA3 pyramidal cells before and after DHPG application (*t* = −1.778, *p* = .136).

**FIGURE 2:**
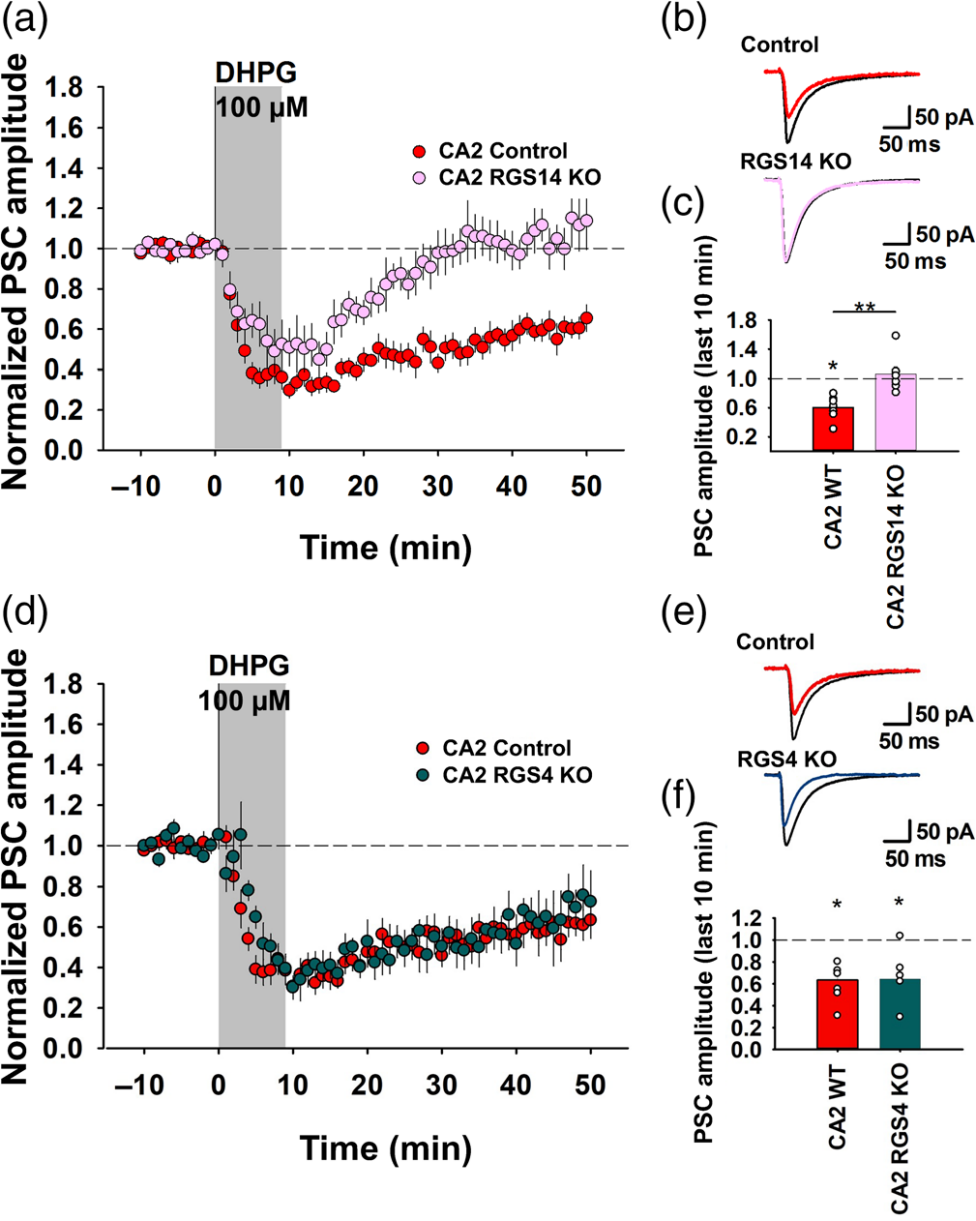
RGS14, but not RGS4, is required for group I mGluR LTD in CA2. (a) Normalized PSC amplitude following 100 μM DHPG application. Red circles represent PSC amplitude of CA2 pyramidal cells from C57BL/6J wildtype (WT) control mice (*n* = 8). Pink circles represent PSC amplitude of CA2 pyramidal cells in RGS14 KO mice (*n* = 7). Gray shaded area indicates duration of DHPG application. (b) Example PSC traces from CA2 pyramidal cell before and after DHPG application. Black traces represent PSC response before DHPG application. Above: red trace indicates PSC response after DHPG application in CA2 cell from a WT control mouse. Below: pink trace represents response after DHPG application from an RGS14 KO mouse. (c) Normalized PSC amplitude in CA2 WT control (red) and RGS14 KO mice (pink) at 41–50 min from drug onset. White circles represent mean PSC amplitude from individual cells. Single asterisks denote significant difference in PSC amplitude when compared with baseline (*p* < .05). Double asterisks denote significant difference in normalized PSC amplitude when comparing WT control (red) and RGS14 KO (pink) mice. Panels d–f as in a–c except with recordings from CA2 pyramidal cells from WT control mice (red, *n* = 6) and RGS4 KO mice (green, *n* = 5).

Both RGS4 and RGS14 are highly expressed in area CA2 (Lee et al., 2010; Lein et al., 2007), but whether they play a role in mGluR‐LTD is unknown. RGS4 is the only RGS protein known to (negatively) regulate group I mGluRs (Saugstad et al., 1998), and so we predicted that mGluR‐LTD could be enhanced in the RGS4 KO mice. Similarly, we predicted that RGS14 KO mice might also have enhanced mGluR‐LTD, although by a different mode of action; RGS14 has been shown to inhibit MAPK signaling (Foster et al., 2021; Shu et al., 2010) and there is evidence supporting a role for MAPK signaling in mGluR‐LTD in CA1 (Gallagher et al., 2004). To test whether either of these RGS proteins regulate mGluR‐LTD, we performed experiments in slices from either RGS14 or RGS4 KO animals and compared these to experiments performed on slices from WT control mice (C57BL/6J). As in the first set of experiments, application of 100 μM DHPG resulted in a robust LTD of the synaptic responses in slices from controls (WT: 0.6 ± 0.05, *n* = 8, *p* = .001; Figure 2a–c). However, in slices from RGS14 KO animals, DHPG caused only a temporary depression that recovered within 15 min of DHPG discontinuation (KO: 1.07 ± 0.09, *n* = 7, *p* = .423; Figure 2a–c). To determine whether a different RGS protein highly expressed in CA2 also regulated mGluR‐LTD, we repeated the above experiments in RGS4 KO mice and a separate cohort of C57BL/6J WT control mice. Under these same conditions, DHPG caused a similar amount of depression in slices taken from both RGS4 KO (0.65 ± 0.11, *n* = 5, *p* = .005; Figure 2d–f) and control animals (0.63 ± 0.05, *n* = 6, *p* = .038; Figure 2d–f). Thus, contrary to our predictions, knockout of neither RGS4 nor RGS14 resulted in enhanced mGluR‐LTD. Indeed, RGS14 deficiency prevented lasting mGluR‐LTD in CA2 pyramidal cells.

**FIGURE 3:**
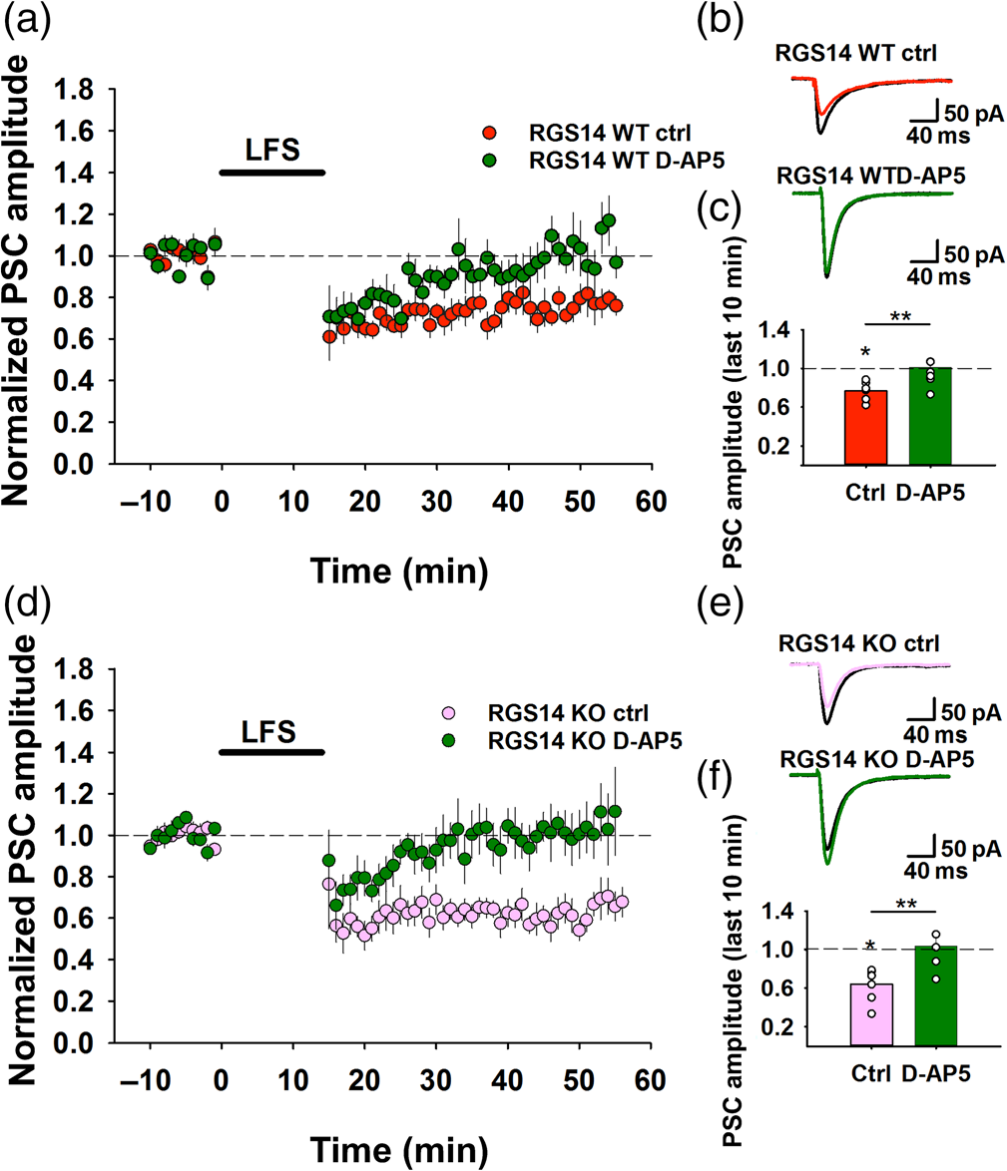
NMDAR‐LTD in CA2 is unaffected by RGS14 knockout. (a) Normalized PSC amplitude following Low Frequency Stimulation (LFS; 900 pulses at 1 Hz, indicated by the bar). Red circles represent PSC amplitude of CA2 pyramidal cells from WT control mice (*n* = 6). Green circles represent PSC amplitude of CA2 pyramidal cells in WT control mice with inclusion of D‐AP5 in the bath (*n* = 7). (b) Example PSC traces from CA2 pyramidal cell before and after LFS. Black traces represent PSC response before LFS. Above: red trace indicates PSC response after LFS in a CA2 cell. Below: green trace represents response after LFS with D‐AP5. (c) Normalized PSC amplitude in CA2 WT control mice without D‐AP5 (red) or with D‐AP5 (green) at 46–55 min from LFS onset. White circles represent mean PSC amplitude from individual cells. Single asterisks denote significant difference in PSC amplitude when compared with baseline (*p* < .05). Double asterisks denote significant difference in normalized PSC amplitude when comparing without (red) and with (green) D‐AP5. Panels d–f as in a–c except with recordings from CA2 pyramidal cells from RGS14 KO mice without D‐AP5 (pink, *n* = 7) and RGS4 KO mice with D‐AP5 (green, *n* = 5).

Given this dependence of CA2 mGluR‐LTD on RGS14, and the role of RGS proteins in regulating G‐protein signaling, we wondered whether RGS14 was required only for mGluR‐LTD, or whether another form of LTD also involving glutamate receptor internalization similarly required RGS14. We, therefore, examined whether low frequency stimulation‐induced LTD (LFS‐LTD), which is not thought to be G‐protein dependent, is affected by the absence of RGS14. We found that LFS (LFS; 900 pulses delivered at 1 Hz; (Dudek & Bear, 1992)) induced LTD in slices from both control (0.77 ± 0.04, *n* = 6; Figure 3a,b) and RGS14 KO mice (0.64 ± 0.06, *n* = 7; Figure 3d,e). LFS‐LTD is NMDA receptor‐dependent in region CA2 in both control and RGS14 KOs, as demonstrated by the finding that the NMDAR antagonist APV was effective at inhibiting LTD in both cases (RGS14 WT: 1.01 ± 0.09, *n* = 7: RGS14 KO 1.04 ± 0.13, *n* = 5; Figure 3a–f). These results indicate that RGS14 is dispensable for CA2 LFS‐LTD which is dependent on NMDARs, but is required for mGluR‐LTD in CA2.

### 3.2 mGluR LTD in CA2 requires STEP

**FIGURE 4:**
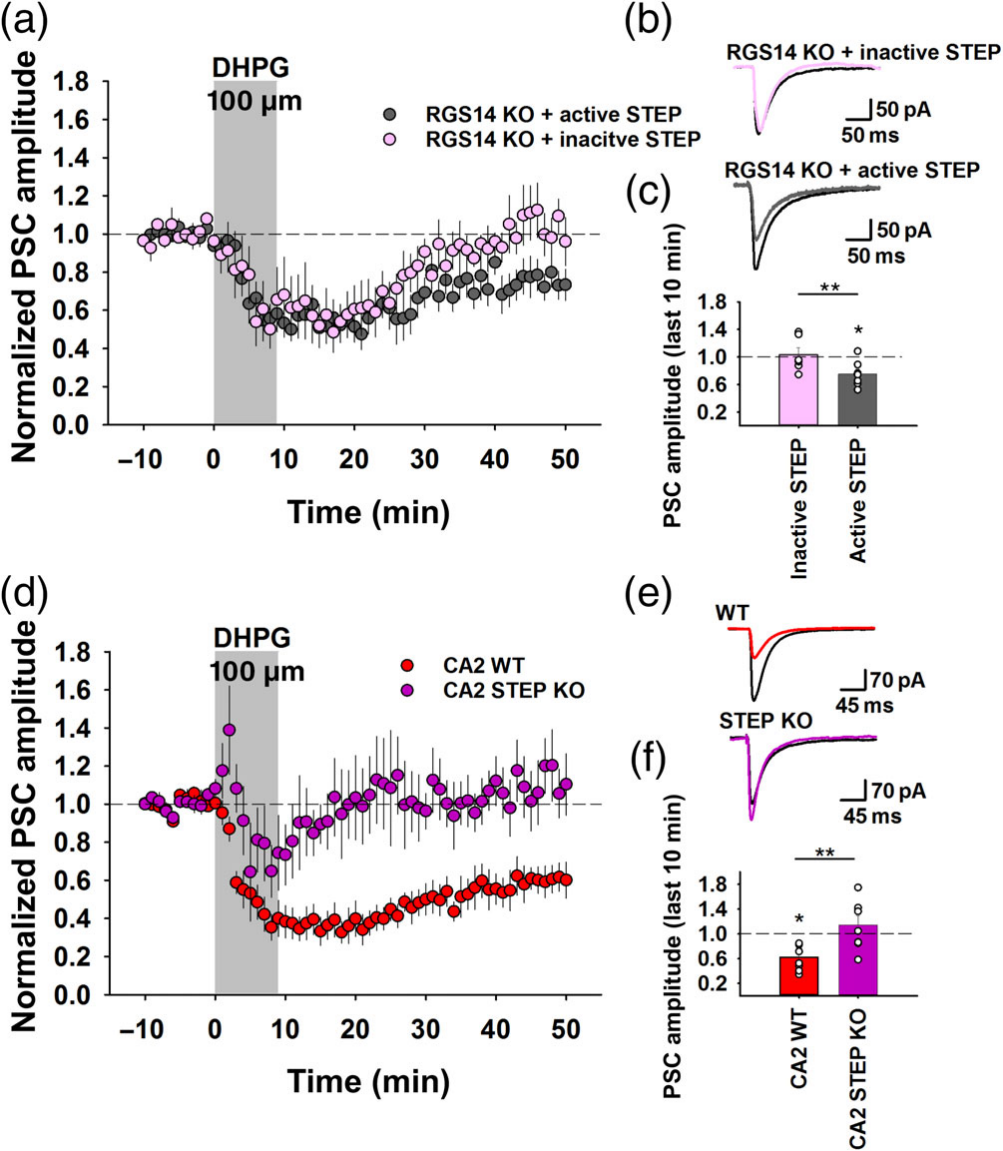
mGluR LTD in CA2 requires STEP. (a) Normalized PSC amplitude following 100 μM DHPG application. Pink circles represent PSC amplitude of CA2 pyramidal cells from RGS14 KO mice with inactive STEP in the intracellular filling solution (*n* = 6). Gray circles represent PSC amplitude of CA2 pyramidal cells from RGS14 KO mice with active STEP in intracellular filling solution (*n* = 7). Gray shaded area indicates duration of DHPG application. (b) Example PSC traces from a CA2 pyramidal cell before and after DHPG application. Black traces represent PSC response before DHPG application. Above: Pink trace indicates PSC response after DHPG application in an RGS14 KO mice with inactive STEP. Below: Gray traces represents response after DHPG application in an RGS14 KO mice with active STEP in intracellular solution. (c) Normalized PSC amplitude in RGS14 KO (pink) and RGS14 KO with STEP in intracellular solution (gray) at 41–50 min after onset of DHPG application. White circles represent mean PSC amplitude from individual cells. Single asterisks denote significant difference in PSC amplitude when compared with baseline (*p* < .05). Double asterisks denote significant difference in normalized PSC amplitude when comparing RGS14 KO with inactive STEP with RGS14 KO with active STEP in the intracellular solution. Panels d–f as in a–c except with recordings from CA2 pyramidal cells from WT control mice (red, *n* = 7) and STEP KO mice (magenta, *n* = 8). Double asterisks denote significant difference in normalized PSC amplitude when comparing data from WT control mice with data from STEP KO mice.

The findings presented above demonstrate that RGS14 is required for mGluR‐LTD in CA2 pyramidal cells, but whether it plays a direct role in this form of plasticity is unknown. We therefore investigated other possible mechanisms that might underlie this deficit. Previous work has demonstrated that Striatal‐Enriched Phosphatase (STEP) is required for mGluR LTD in CA1 region of hippocampus (Zhang et al., 2008), and so we examined whether STEP was similarly involved in mGluR LTD in area CA2. As protein dephosphorylation via STEP is another reported mediator of mGluR‐LTD in CA1 (Gladding et al., 2009), we first tested whether enhancing STEP activity could overcome deficiencies in DHPG‐induced mGluR‐LTD in slices from RGS14 KO mice. To do this, we included purified active or inactive STEP protein in the intracellular filling solution. Indeed, we found that inclusion of dephosphorylated, active STEP in the patch pipette apparently rescued mGluR‐LTD in the RGS14 KO tissue in CA2 (0.74 ± 0.07, *n* = 7; Figure 4a–c). A *t*‐test using raw PSC amplitudes before and after DHPG application revealed a significant difference in PSC amplitude at baseline compared with 41–50 min into recovery (*t* = −3.083, *p* = .022). When comparing normalized PSC amplitude in the RGS14 KO mice following DHPG application, we found a significant difference when active STEP was included in the intracellular solution compared with when inactive STEP was included (Figure 4a–c; *t* = 2.288, *p* = .043). Furthermore, no statistically significant difference was found when comparing PSC amplitude after DHPG of RGS14 KO mice with active STEP to previous WT experiments (*t* = −1.577, *p* = .139). These data strongly suggest that mGluR‐LTD impairment accompanying RGS14 deficiency can be overcome by supplementing with active STEP.

We next asked whether STEP KO would mimic RGS14 KO in preventing mGluR‐LTD in CA2. We found no lasting depression in CA2 induced by application of DHPG in hippocampal slices from STEP KO mice (1.15 ± 0.14, *n* = 8, *p* = .929) whereas DHPG readily induced depression in control CA2 neurons (0.63 ± 0.08, *n* = 7, *p* = .01; Figure 4d–f).

**FIGURE 5:**
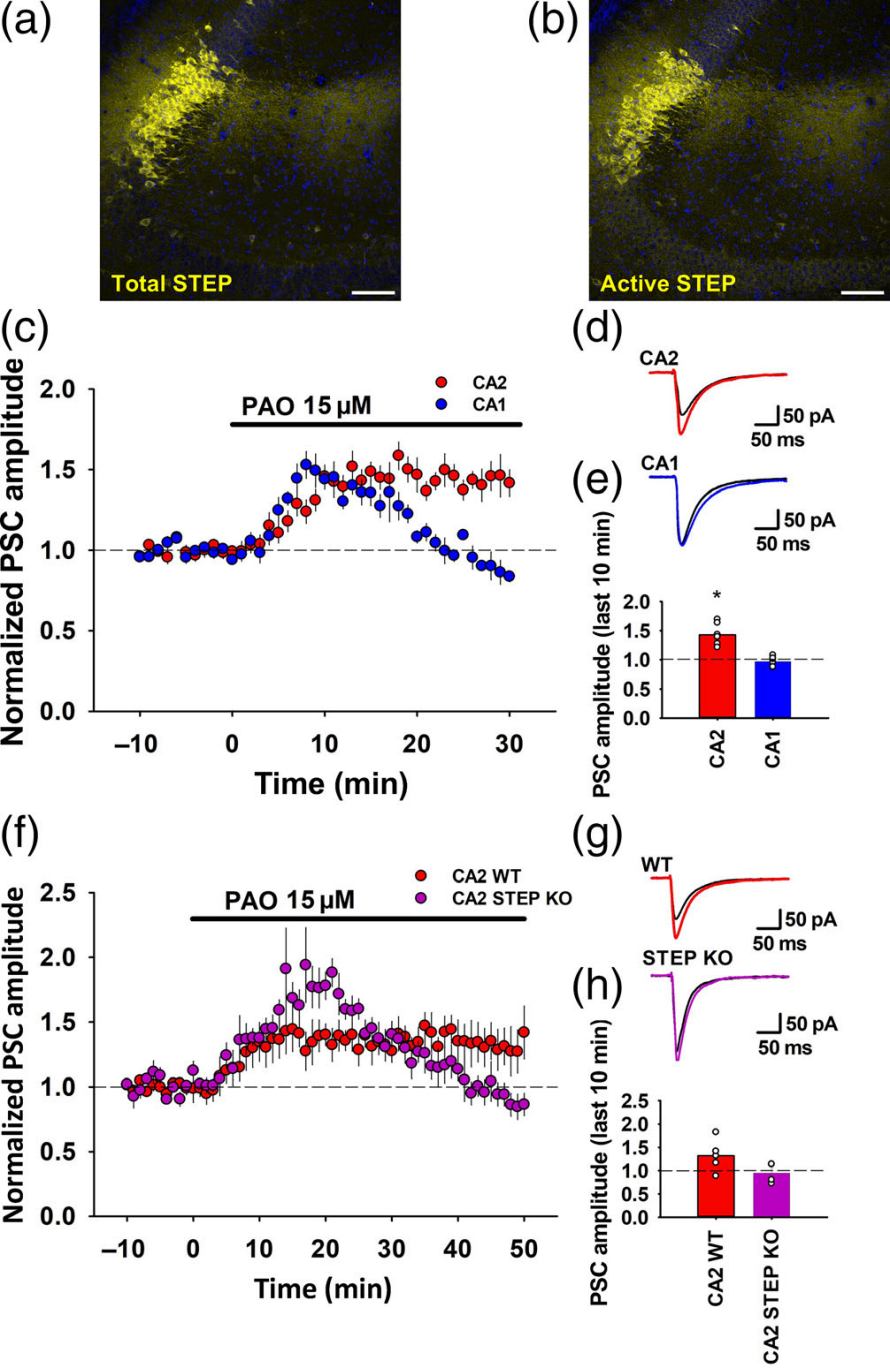
STEP is constitutively active and can regulate synaptic strength in CA2. (a,b) Coronal sections from a C57BL/6J mouse showing the hippocampus stained with total STEP or with active, non‐phosphorylated STEP. Scale bars = 100 μm. (c) Normalized PSC amplitude following 15 μM PAO application. Data points represent the mean PSC amplitude. Error bars represent the standard error of the mean. Red circles represent PSC amplitude of CA2 pyramidal cells (*n* = 8) and blue circles represent PSC amplitude of CA1 pyramidal cells (*n* = 6) from C57BL/6J mice. Black line indicates duration of PAO application. (d) Example PSC traces from CA2 and CA1 pyramidal cells before and after PAO application. Black traces represent PSC response before PAO application. Above: Red trace indicates PSC response of CA2 cell after 30 min of PAO application. Below: Blue trace represents response of CA1 cell after PAO application. (e) Normalized PSC amplitude in CA2 (red) and CA1 (blue) at 21–30 min after start of PAO. White circles represent mean PSC amplitude from individual cells. Single asterisk denotes significant difference when compared with baseline (*p* ≤ .05). Panels f–h as in c–e, but with recordings from CA2 pyramidal cells from WT control and STEP KO mice. Red circles represent PSC amplitude of CA2 pyramidal cells from WT control mice (*n* = 5). Magenta circles represent PSC amplitude of CA2 pyramidal cells from STEP KO mice (*n* = 4).

Immunohistochemical staining for STEP protein is very high in CA2 relative to other hippocampal areas (Boulanger et al., 1995), and this is also the case for the active, non‐phosphorylated STEP (Figure 5a,b). This suggests that the majority of STEP is constitutively active in CA2 pyramidal cells and may therefore be regulating synaptic transmission and electrophysiological properties under baseline conditions, in addition to regulating activity‐dependent synaptic plasticity. To investigate whether STEP plays any role in regulating baseline synaptic transmission in CA2, we applied a tyrosine phosphatase inhibitor phenylarsine oxide (PAO; Garcia‐Morales et al., 1990) to CA2 or CA1 neurons in the bath ACSF solution. We found that 15 μM PAO rapidly induced potentiation of synaptic responses in both CA2 and CA1 pyramidal cells. Differences emerged, though, in the continued presence of the drug: PSC amplitudes recorded in CA1 returned to baseline levels within 30 min (0.97 ± 0.03, *n* = 6; Figure 5c–e), and in some cases “undershot” the baseline upon longer incubation. In contrast, the increase in PSC amplitude remained in CA2 (1.46 ± 0.06, *n* = 8). Paired *t*‐test of raw PSC amplitude before and after PAO application revealed a significant difference in CA2 responses at 21–30 min into PAO application (*t* = 6.767, *p* ≤ .001) but not in CA1 (*t* = −1.249, *p* = .267). A *t*‐test using normalized PSC amplitudes comparing CA2 and CA1 responses at 21–30 min into PAO application revealed a significant difference between CA2 and CA1 (*t* = 6.278, *p* ≤ .001; Figure 5e).

To determine whether this increase in PSC amplitude after PAO application was due to active STEP rather than another tyrosine phosphatase, PAO was applied to CA2 pyramidal cells in slices from both control and STEP KO mice. In this case as before, we found that PAO induced an initial increase in PSC amplitude of both WT and STEP KO. In CA2 of the STEP KO mice however, PSC amplitudes decreased back to baseline levels at 41–50 min into application (0.95 ± 0.11, *n* = 4), similar to what we observed in CA1 pyramidal cells. This decrease in PSC amplitude was not seen in CA2 of the control slices (1.32 ± 0.15, *n* = 5; Figure 5f–h). Paired *t*‐test comparing raw PSC amplitude at baseline and 41–50  min into PAO application revealed that PSC amplitude had not significantly changed in the WT (*t* = 1.591, *p* = .187) or STEP KO mice (*t* = −0.252, *p* = .817). The *t*‐test of normalized PSC amplitude revealed no significant difference between the two (*t* = 1.862, *p* = .105). Although synaptic currents in CA1 and CA2 differ in their response to tyrosine phosphatase inhibition and the difference appears to be generally mimicked in STEP KO vs. WT CA2, we cannot definitively conclude that the effects of PAO werdue to the inhibition of STEP. However, these studies are consistent with staining showing more non‐phosphorylated, active STEP in CA2 than in CA1.

### 3.3 mGluR‐LTD in CA2 requires protein synthesis

**FIGURE 6:**
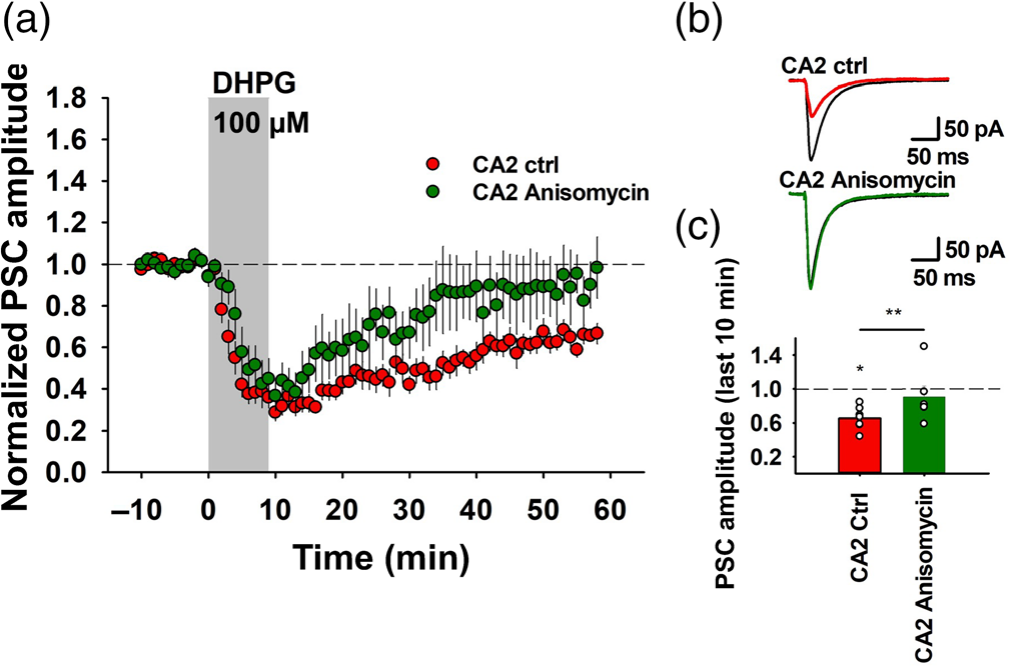
Group I mGluR LTD in CA2 requires protein synthesis. (a) Normalized PSC amplitude following 100 μM DHPG application. Red circles represent PSC amplitude of CA2 pyramidal cells from C57BL/6J mice (*n* = 9). Green circles represent PSC amplitude of CA2 pyramidal cells in the presence of protein synthesis inhibitor anisomycin (*n* = 6). Gray shaded area indicates duration of DHPG application. (b) Example PSC traces from CA2 pyramidal cell before and after DHPG application. Black traces represent PSC response before DHPG application. Above: Red trace indicates PSC response after DHPG application in CA2 cells with vehicle only. Below: Green traces represents response after DHPG application in the presence of anisomycin. (c) Normalized PSC amplitude in CA2 with either vehicle (0.1% DMSO) (red) or anisomycin (green) at 50–59 min after DHPG onset. Single asterisk denotes significant difference when compared with baseline (*p* ≤ .05). Double asterisks denote significant difference in normalized PSC amplitude when comparing without (red) and with (green) anisomycin.

To further examine the mechanism(s) by which mGluR activation induces LTD in CA2, we investigated the involvement of protein synthesis, another process shown to be important for mGluR LTD in CA1 pyramidal cells (Huber et al., 2000; Zhang et al., 2008). Previous work from our lab has shown that the protein synthesis inhibitors cycloheximide and anisomycin both rapidly elicit a modest decrease in the baseline PSC amplitude in CA2 but not in CA1 pyramidal cells (Farris et al., 2019). With this previous finding in mind, we applied anisomycin at least 20 min before DHPG application to ensure that a new baseline was established after the decrease in PSC amplitude, and continued drug exposure during and after DHPG application (60 min total; Figure 6). Although treatment with anisomycin had negligible effects on the immediate response to DHPG, mGluR‐LTD eventually decayed back to baseline values over time (0.9 ± 0.13, *n* = 6). A *t*‐test of raw PSC amplitude before and after DHPG with anisomycin revealed no significant difference (*t* = −1.036, *p* = .348), although application of DHPG induced a significant depression in PSC amplitude when hippocampal slices were incubated in vehicle control solution (0.65 ± 0.04, *n* = 9, *t* = −6.087, *p* ≤ .001). Paired *t*‐test comparing normalized PSC amplitude at 50–59 min with or without anisomycin revealed a significant difference (*t* = −2.19, *p* = .047; Figure 6c), indicating that mGluR‐LTD in CA2 pyramidal cells is similar to the mGluR‐LTD characterized previously in CA1 in its dependence on new protein synthesis (Huber et al., 2000; Zhang et al., 2008).

### 3.4 CaMKII inhibition does not rescue LTD in RGS14 KO

Recent investigations have sought to identify the proteins that interact with RGS14 (Evans, Gerber, et al., 2018). By immunoprecipitating RGS14 from mouse brain homogenates, Evans, Parra‐Bueno, et al. (2018) identified several novel RGS14 binding partners that have been implicated in synaptic transmission and plasticity. This includes proteins such as synapsin‐2, a regulator of neurotransmitter release (Sugiyama et al., 2000), elongation factor 1 alpha, important for tRNA delivery to the ribosome during protein synthesis‐dependent plasticity (Huang & Hsu, 2006), and microtubule‐associated proteins Map1b and Map2, important for the regulation of dendritic spine morphology (Jaworski et al., 2009; Tortosa et al., 2011). One of the proteins that strongly interacts with RGS14 is CaMKII (Evans, Gerber, et al., 2018). Given that CaMKII signaling is crucial for LTP (Lisman et al., 2002; Tao et al., 2021), it is possible that RGS14 is suppressing LTP in CA2 by inhibiting CaMKII signaling. Indeed, a CaMKII inhibitor was shown to inhibit LTP in CA2 of RGS14 KO mice (Evans, Parra‐Bueno, et al., 2018). Given also that such inhibitors of CaMKII have been shown to enhance group I mGluR‐dependent LTD in CA1 (Schnabel et al., 1999), it is also possible that regulation of CaMKII by RGS14 is important not only for suppressing LTP in CA2 but also for regulating other forms of plasticity such as mGluR‐LTD (Foster et al., 2021). Thus, we sought to determine whether CaMKII was in some way modulating mGluR‐LTD via RGS14.

**FIGURE 7:**
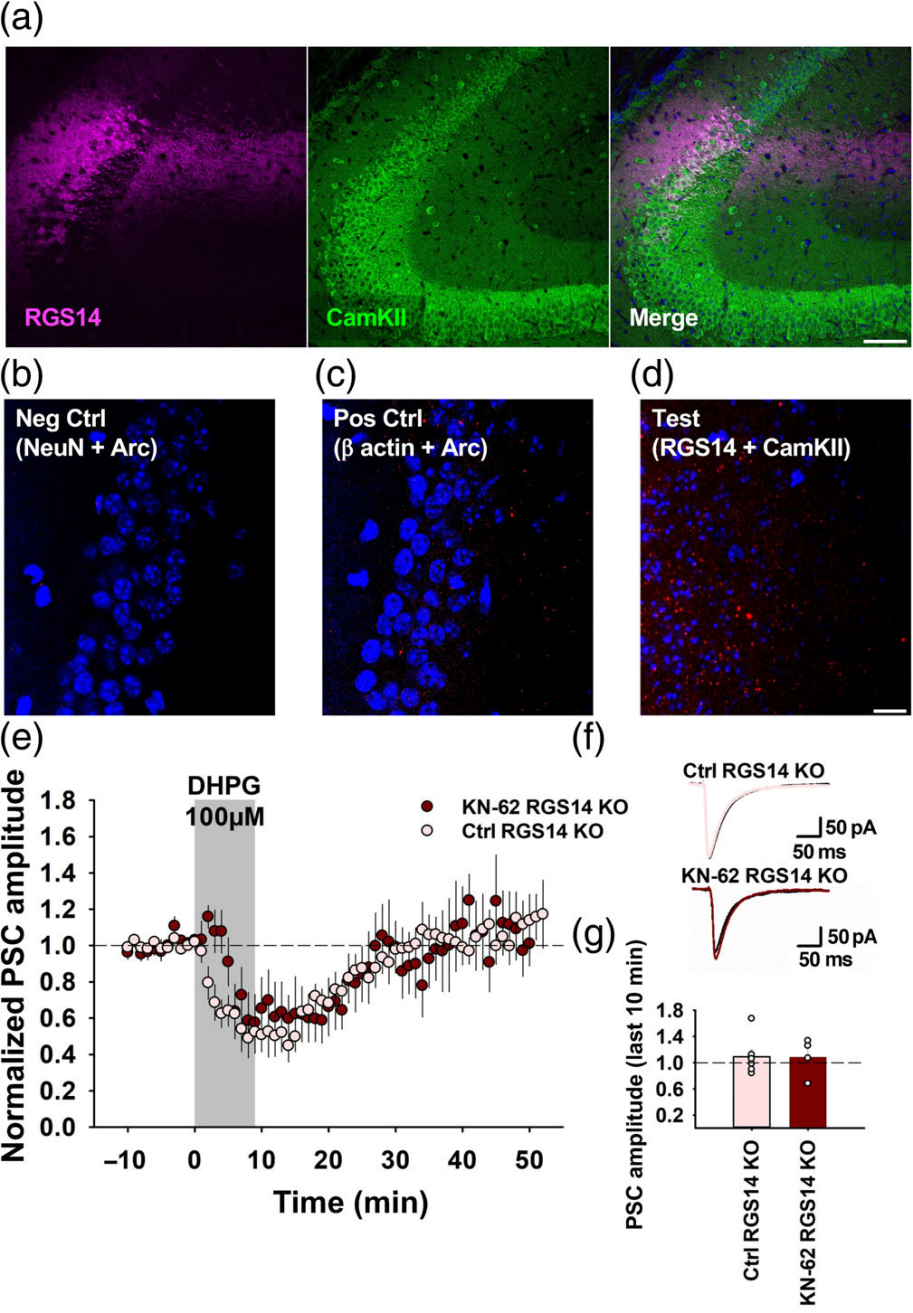
CaMKII interacts with RGS14, but does not prevent mGluR‐LTD in RGS14 KO. (a), Coronal section from a C57BL/6J mouse showing the hippocampus co‐stained with RGS14 (pink) and CaMKII alpha (green). The merged image is shown on the right. Scale bar = 100 μm. (b–d) Coronal section from a C57BL/6J mouse showing duolink staining where signal (red) denotes interaction between two proteins targeted with primary antibodies: (b) negative control using primary antibodies targeting NeuN and Arc (proteins with minimal interaction); (c) positive control primary antibodies targeting β‐actin and Arc (proteins known to interact); and (d), using primary antibodies targeting RGS14 and CaMKII to test for protein interaction. Images in b and c taken from the CA1 region of hippocampus, and the image in d was taken from the CA2 region. Scale bar = 25 μm. (e) Normalized PSC amplitude following 100 μM DHPG application. Pink circles represent PSC amplitude of CA2 pyramidal cells in RGS14 KO mice (*n* = 7). Brown circles represent PSC amplitude of CA2 pyramidal cells in RGS14 KO mice with 10 μM of the CaMKII inhibitor KN‐62 in the bath (*n* = 4). Gray shaded area indicates duration of DHPG application. (f) Example PSC traces from a CA2 pyramidal cell before and after DHPG application. Black traces represent PSC response before DHPG application. Above: Pink trace indicates PSC response after DHPG application in the RGS14 KO mice. Below: Brown trace is a response after DHPG application in the RGS14 KO mice with KN‐62. (g) Normalized PSC amplitude in RGS14 KO (pink) and RGS14 KO with KN‐62 (brown) at 41–50 min after DHPG onset. White circles represent mean PSC amplitude from individual cells.

Importantly CaMKII is expressed throughout the hippocampus, including in CA2 pyramidal cells where RGS14 is expressed (Figure 7a). To determine whether we could detect evidence of a RGS14‐CaMKII protein–protein interaction in brain sections, replicating the findings of Evans, Parra‐Bueno, et al. (2018), we used a PLA. For the negative control, we used an antibody against NeuN together with an antibody to activity regulated cytoskeletal associated protein (Arc). Arc protein is highly expressed in the cytoplasm and is not an interacting partner for the nuclear transcription factor NeuN (Lyford et al., 1995); thus, we anticipated minimal PLA signal between NeuN and Arc antibodies. Using this combination of primary antibodies, we found no apparent signal (Figure 7b), in contrast to our positive control using primary antibodies targeting Arc and β‐actin in CA1 which are known to interact (Figure 7c) (Lyford et al., 1995).

Using primary antibodies raised against RGS14 and CaMKII, we observed an intense PLA signal in CA2 that resembled, and in fact exceeded, the intensity of the positive control. These images are suggestive of an RGS14‐CaMKII interaction, or at least that the proteins are localized within 40 nm of each other in CA2 cell bodies and dendrites (Bagchi et al., 2015; Figure 7d). As no signal was observed when RGS14 or CaMKII primary antibodies were used alone, these data confirm the specificity of the in situ RGS14–CaMKII interaction as previously reported (Evans, Gerber, et al., 2018).

If CaMKII activity is normally suppressed by RGS14, and RGS14 deficiency disinhibits CaMKII, it is possible that the absence of mGluR‐LTD in RGS14 KO mice is driven by hyperactivity of the CaMKII, which promotes/underlies LTP induction. In this case, we hypothesized that the mGluR‐LTD could be rescued in the RGS14 KO with pharmacological inhibitors of CaMKII, which has been shown to enhance DHPG‐induced LTD in CA1 (Schnabel et al., 1999), but see Mockett et al. (2011) and Bernard et al. (2014). We, therefore, applied DHPG together with the CaMKII inhibitor KN‐62 (10 μM) in experiments using the RGS14 KO mice. We found even with a pre‐incubation with KN‐62, DHPG still did not induce LTD in the RGS14 KO tissue (1.08 ± 0.15 PSC amplitude normalized to baseline, *n* = 4; Figure 7e–g). A *t*‐test using raw PSC amplitude comparing baseline and responses at 41–50 min following DHPG onset revealed no significant difference in PSC amplitude before and after DHPG application (*t* = 1.133, *p* = .339). Comparing normalized PSC amplitude in the RGS14 KO mice with and without the CaMKII inhibitor KN‐62 (at 41–50 min), again, revealed no significant difference between the two conditions (*t* = 0.119, *p* = .908). These data argue against the idea that the lack of mGluR‐LTD in RGS14 KO is due to unrestrained CaMKII activity. Note that these experiments have not addressed the alternative hypothesis that CaMKII activity facilitates mGluR‐LTD in CA2 (Bernard et al., 2014; Mockett et al., 2011). Future studies will be needed to further investigate this possibility.

### 3.5 Social discrimination is impaired in RGS14 KO mice

**FIGURE 8:**
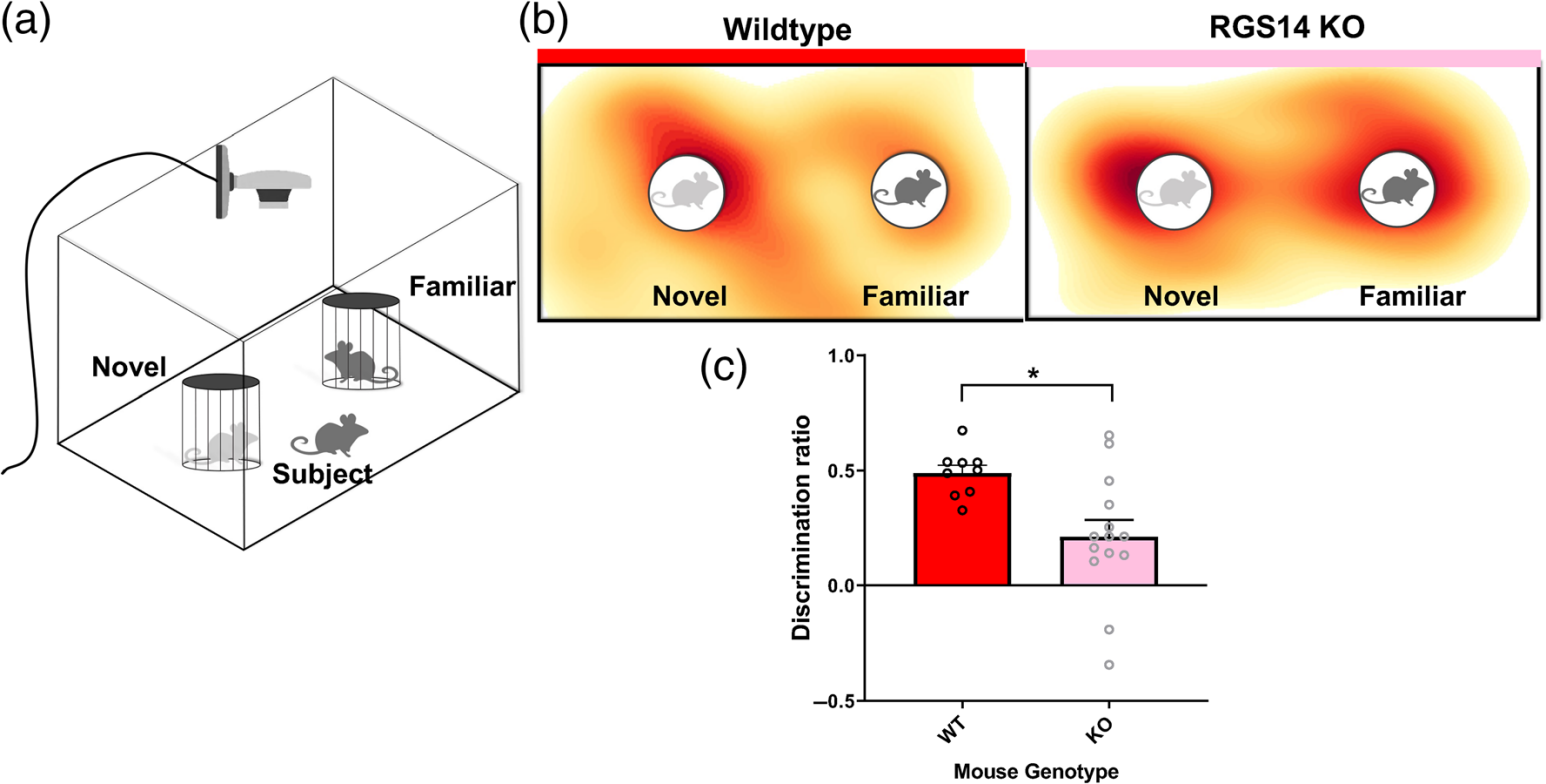
Social discrimination is impaired in RGS14 KO mice. (a) Illustration showing the experimental layout. Mice are presented with either a novel mouse or a familiar cagemate and allowed to freely investigate within the arena. (b) Example heatmaps from an individual WT control mouse or an RGS14 KO mouse. Red represents greater time spent interacting. (c) Discrimination ratios from WT control mice (red) and RGS14 KO mice (pink). White circles represent discrimination ratios from individual animals. Asterisk denotes significant difference between WT control and RGS14 KO (*p* ≤ .05).

CA2 has been shown to be necessary for a number of social behaviors in mice including aggression and social recognition memory (Hitti & Siegelbaum, 2014; Leroy et al., 2018; Pagani et al., 2015; Smith et al., 2016), and group I mGluRs have been linked to sociability (Chung et al., 2015; Mesic et al., 2015). Thus, it is possible that mGluR‐dependent plasticity in CA2 is important for social memory. Therefore, given our results showing that RGS14 KO disrupts mGluR LTD in CA2, we investigated the impact of genetic RGS14 deficiency in a social recognition memory task, where mice were allowed to freely explore an arena containing both novel and familiar conspecifics (Figure 8). As expected, WT control animals were able to discriminate between novel and familiar mice, showing a preference for interacting with the novel mouse compared with the familiar cage mate (one sample *t*‐test, *p* < .001; Figure 8). In contrast, RGS14 KO animals spent similar amounts of time investigating novel and familiar mice (one sample *t*‐test, *p* = .207), resulting in a significantly lower discrimination ratio than control animals (*t*_16_ = 3.185, *p* = .013; Figure 8). Total interaction time was not different between control and RGS14 KO animals (*t*_16_ = 0.593, *p* = .561; data not shown), suggesting no differences in overall sociability. This difference in social discrimination is typically interpreted as a loss of social recognition memory in the RGS14 KO mice, but it may also be interpreted as a loss of preference for novel to familiar social stimuli. These data show that in addition to playing a role in spatial and fear memory (Alexander et al., 2019; Lee et al., 2010), RGS14 is also important for social recognition memory. Further studies using a targeted knockout of RGS14 specifically in CA2 pyramidal neurons will be required to better implicate RGS14 in CA2 in these types of behavioral tasks.

## 4 DISCUSSION

Our experiments show that DHPG induces robust depression in CA2 pyramidal neurons. DHPG did not induce lasting depression in slices from RGS14 KO mice but did in RGS4 KO mice, suggesting that RGS14, but not RGS4, is required for mGluR‐LTD in CA2. The result was surprising in that RGS14 is thought to specifically target Gα_i/o_ coupled G‐protein coupled receptors (GPCRs) (Brown et al., 2016; Vellano et al., 2013) and group I mGluRs are coupled to Gα_q_ (Gladding et al., 2009; Saugstad et al., 1998). RGS14 has a number of functional domains/motifs that are distinct from its RGS domain, so RGS14 actions on mGluR‐LTD could be independent of its GAP activity. Consistent with this idea, RGS14 contains a GPR motif that is required for plasma membrane anchoring (Brown et al., 2015), and this motif was found to be essential for RGS14 regulation of synaptic plasticity in hippocampal neurons independent of the RGS domain function (Squires et al., 2021). Additionally, RGS14 binds active H‐Ras to inhibit ERK signaling (Shu et al., 2010). In contrast, RGS4 is a small protein that lacks these other scaffolding domains and functions. As we found that DHPG‐induced LTD in slices from RGS4 KO mice was similar to that in WT mice, we conclude that RGS4 does not play a critical role in mGluR‐LTD in CA2, despite previous work showing that it blocks mGluR and Gq‐mediated effects in CA1 hippocampal neurons (Saugstad et al., 1998).

Previous work on activity‐dependent LTD (450 pulses at 2 Hz) in rat CA2 had shown that LTD is expressed in only ~62% of CA2 pyramidal cells (Zhao et al., 2007). In the present study, however, we observed that LFS could induce LTD in the overwhelming majority of CA2 neurons in mice (Figure 3). Because NMDA receptor‐dependent LTD induced by LFS is normal in slices from RGS14 KO mice (Figure 3), we propose that RGS14 is not likely to be directly regulating core trafficking machinery necessary for glutamate receptor endocytosis, which would be common to postsynaptically expressed LTD regardless of how it is induced. However, further experiments would be needed to rule out a requirement for RGS14 in endocytic processes. Although our experiments do not implicate or rule out presynaptic mechanisms of LTD in CA2 like that mediated by endocannabinoids (Gerdeman et al., 2002), we note that *Cnr1* is highly expressed in CA2 neurons, like in CA1 (Farris et al., 2019; Lein et al., 2007). Thus an important follow‐up to these studies will be to investigate whether CB1‐dependent LTD is found in CA2.

Earlier work demonstrated that LTP could be induced in CA2 neurons from RGS14 KO mice, unlike in CA2 neurons from control mice (Evans, Parra‐Bueno, et al., 2018; Lee et al., 2010). Thus, while we cannot determine whether the behavioral effects of RGS14 knockout are due to the mGluR‐LTD loss, or from LTP gain in CA2, it appears that RGS14 knockout shifts this balance away from LTD and toward LTP in CA2. Thus RGS14 is normally required for some forms of synaptic plasticity (mGluR‐LTD), while suppressing others (LTP).

The results of our experiments show that the group I mGluR agonist DHPG induces synaptic depression in CA2 to a greater degree than we see in CA1 and CA3. Although we did not observe significant LTD outside of area CA2, we note that our experiments were carried out at room temperature while previous studies were closer to physiological temperatures (Fitzjohn et al., 1999). Because the rate of receptor internalization in these types of experiments is certainly temperature dependent (Delvendahl et al., 2016; Weigel & Oka, 1981), this difference between subregions may, or may not, exist naturally, in vivo. In either case though, our experimental conditions revealed a difference between CA2 and the other hippocampal subfields, which was reflective of RGS14 expression and the amount of labeled phosphoinositide accumulation in response to a mGluR agonist (Hwang et al., 1990; Lee et al., 2010).

In the brain, STEP activity is regulated by the phosphorylation of serine residues (Serine^49^ and Serine^221^) by PKA, which renders it inactive by inhibiting STEP from binding to its substrates (Paul et al., 2000). Supporting the idea that STEP activity, not just STEP protein levels, is important for mGluR LTD in CA2 was our data showing that active, but not inactive, STEP was able to rescue mGluR‐LTD in mice lacking RGS14. Although we did not test whether GST‐STEP enhances mGluR‐LTD in WT neurons, we expect that it could be the case; previous work in FMRP null mice has shown that excess STEP in synaptoneurosomes correlates with the exaggerated mGluR‐LTD phenotype in CA1 (Goebel‐Goody et al., 2012). Substrates of STEP include GluN2B NMDA receptors, important for the induction of LTD and LTP (Braithwaite et al., 2006; France et al., 2017; Muller et al., 2009) as well as GluA2 subunits of AMPA receptors (Zhang et al., 2008), which govern calcium permeability of AMPA receptors (Chater & Goda, 2014). Upon dephosphorylation by STEP, both GluN2B and GluA2 receptors can be internalized. In addition to this, STEP also inactivates enzymes such as MAPK/ERK important for plasticity via its roles in stabilizing dendritic spines, initiating local protein synthesis, and regulating nuclear transcription by phosphorylating specific transcription factors such as cAMP response element binding protein (CREB) (Davis et al., 2000; Sweatt, 2004). Thus, it is clear that STEP regulates synaptic transmission and plasticity in a variety of different ways. Nevertheless, the requirement for STEP in mGluR‐LTD in CA2 illustrates that STEP is integral for synaptic plasticity in CA2.

Thus far, work in CA1 manipulating STEP activity has provided some insight into how STEP regulates synaptic plasticity. For example, STEP inhibition increases NMDA receptor mediated currents, demonstrating that STEP negatively regulates NMDA receptors (Pelkey et al., 2002). Consequently, purified STEP inhibited LTP induction (Pelkey et al., 2002). In addition, STEP KO mice (Venkitaramani et al., 2009) have also provided insight into STEP function and its role in cognition; STEP KO mice have enhanced hippocampal‐dependent spatial memory in the Morris water maze (Venkitaramani et al., 2011). These mice also have enhanced dominance behavior and lower seizure thresholds compared with WT mice (Sukoff Rizzo et al., 2014). Thus given the enrichment of STEP in CA2, it is possible that some of these effects observed in STEP KO mice are due to disruption of synaptic plasticity in CA2. Interestingly, RGS14 KO mice also have enhanced learning in the Morris water maze (Lee et al., 2010) as well as the social memory deficits reported here. Although the mice we used in this study are complete knockouts of RGS14, and thus could possibly have developmental effects unrelated to CA2 synaptic plasticity, RGS14 is undectable at birth and increases to adult levels by postnatal day 21, suggesting that embryonic development is likely to be normal (Evans et al., 2014). In mice, RGS14 is remarkably enriched in CA2 and the fasciola cinerea, but it is also expressed elsewhere in the brain, particularly in human and non‐human primates, where it is also expressed in structures such as the basal ganglia and amygdala (Montanez‐Miranda et al., 2022). Thus, one of the limitations of our behavioral experiment is that other brain regions normally expressing RGS14 could be influenced by the knockout—perhaps via loss of mGluR‐LTD or enhanced LTP as in CA2, but perhaps not. Nevertheless, our results are supportive of the idea that synaptic plasticity in CA2, normally favoring LTD, is important for social memory in mice.

In summary, we found evidence that group I mGluR‐LTD is a prominent form of synaptic plasticity in CA2 *stratum radiatum* in P13‐P21 mice. Although additional experiments are needed to support some conclusions as noted above, these data support a model in which a core mGluR signaling pathway is conserved between CA2 and CA1 and elsewhere, but that CA2 contains unique regulatory molecules, including RGS14, which can tune this core signaling pathway to increase the magnitude of LTD. CA2 has been shown to regulate specific functions such as social cognition, and these results highlight a possible role for RGS14 and STEP in CA2‐dependent behaviors associated with silencing CA2. Thus far, several neuropsychiatric disorders have been linked to changes in mGluR‐LTD and STEP activity (Huber et al., 2002; Nosyreva & Huber, 2006). For example, fragile X mental retardation (FMR1) KO mice, a model for the study of fragile X syndrome, have exaggerated mGluR‐LTD and increased STEP expression. Thus it makes sense that genetically reducing STEP levels in the FMR1 KO mice rescues deficits such as the social abnormalities and epileptic activity (Goebel‐Goody et al., 2012). Because RGS14 is enriched in CA2 of human brain, (Carstens et al., 2021; Squires et al., 2018), it is well positioned to modulate synaptic plasticity, and perhaps some forms of social cognition in humans.

## CONFLICT OF INTEREST STATEMENT

The authors declare no relevant conflicts of interest.

## Data Availability

The data that support the findings of this study are available from the corresponding author upon reasonable request.
